# Genome Agnostic Reprogramming of Acute Myelocytic Leukemia Hallmarks by Targeting Non-Oncogene Addictions with Azacitidine Plus Pioglitazone and All-Trans Retinoic Acid

**DOI:** 10.3390/ijms27021067

**Published:** 2026-01-21

**Authors:** Dennis Christoph Harrer, Florian Lüke, Tobias Pukrop, Albrecht Reichle, Daniel Heudobler

**Affiliations:** 1 Department of Internal Medicine III, Hematology and Oncology, University Hospital Regensburg, DE-93053 Regensburg, Germany; 2 Division of Personalized Tumor Therapy, Fraunhofer Institute for Toxicology and Experimental Medicine, DE-93053 Regensburg, Germany; 3 Bavarian Cancer Research Center (BZKF), University Hospital Regensburg, DE-93053 Regensburg, Germany

**Keywords:** relapsed/refractory acute myelocytic leukemia (AML), leukemia hallmarks as therapeutic target, non-oncogene addictions, differentiation, immuno surveillance, inflammation, metabolism, anakoinosis, pioglitazone, all-trans retinoic acid, azacitidin

## Abstract

The search for new therapeutic principles is essential for treating relapsed/refractory (r/r) acute myeloid leukemia (AML). Novel principles include genome-agnostic differentiation induction, controlling AML-triggering inflammation, potentiating the immune response and ‘normalizing’ AML metabolism. This review summarizes data from a phase I study (10 patients, pts) and three case reports reporting 7 pts on the treatment of r/r AML by reprogramming AML hallmarks using APA, low-dose azacitidine, pioglitazone (PPARα/γ agonist) and all-trans retinoic acid. APA reprograms the r/r AML phenotype in patients with clinically and molecularly/genetically unfavorable risk profiles (17 pts, 16 refractory, one relapsed) in a genome-agnostic manner, restoring the plasticity of AML hallmarks, thereby improving immune surveillance, attenuating inflammation-triggered promotion of AML and distant microbial inflammation (healing of fungal pneumonia during induction of complete remission (CR) with APA), while normalizing leukemia metabolism (restoring phagocytosis and ROS production in leukemic neutrophils). APA induces CR in 10 pts (59%), with only modest hematotoxicity following CR induction. This allows treatment to be carried out in an outpatient setting, including for elderly and comorbid patients. Triple transcriptional modulation, facilitated by epigenetic modelling with azacitidine, targets reprogramming of non-oncogene addiction networks in AML, re-establishing functionally active, closely interrelated myeloid hallmarks and AML cell death genome-agnostically.

## 1. Introduction

Despite the steadily growing portfolio of approved drugs, including classic cytotoxic therapies such as CPX351 (liposomal encapsulation of cytarabine/daunorubicin at a molar ratio of 5:1), immunotherapies such as improved techniques of allogeneic hematopoietic stem cell transplantation (allo-HSCT) and CAR T cell therapy, as well as chemo-immunoconjugates such as gemtuzumab ozogamicin (a CD33 antibody–drug conjugate) and drugs targeting non-oncogene addiction (NOA) targets for large-scale reprogramming AML (such as hypomethylating agents (HMAs), 5-azacytidine (azacitidine) administered intravenously or orally or 2-deoxy-5-azactidine (decitabine), remission induction and maintenance therapy in acute myeloid leukemia (AML) for relapsed or refractory (r/r) AML is still challenging. Besides HMAs, which initiate epigenetic modelling and derepression of tumor suppressor genes, further drugs targeting NOAs are venetoclax (a BCL2 inhibitor) and glasdegib (a hedgehog inhibitor). A specific group of drugs directed towards oncogene addictions, whose clinical activity depends on the presence of recurrent aberrations, includes midostaurin, gilteritinib and quizartinib (FMS-like tyrosine kinase 3 inhibitors), ivosidenib, olutasidenib and enasidenib (isocitrate dehydrogenase 1 and 2 inhibitors), and others [1,2,3,4,5,6,7,8,9,10,11,12,13,14,15,16,17].

The search for novel therapeutic principles for treating r/r AML is imperative, including exploring mechanisms for inducing differentiation, controlling inflammation, potentiating the immune response, and normalizing AML metabolism to prevent the rapid repopulation of blasts [18,19,20,21,22,23].

At first glance, the unique functional defect acquired by AML blasts, the inability to differentiate into neutrophils under disease conditions, may be at the center of attention. However, the question of how systemic AML therapy may induce genome-agnostic differentiation in non-acute promyelocytic leukemia (non-PML) AML remains widely unanswered. Furthermore, how are the differential molecular rationalizations for differentiation induction organized in commonly genetically heterogeneous r/r non-PML AML [17,24,25]?

Similar to ATRA or arsenic trioxide in PML, isocitrate dehydrogenase 1 and 2 inhibitors, menin inhibitors and FMS-like tyrosine kinase 3 inhibitors induce differentiation in AML in an oncogene-addicted way [12,26]. Differentiation in non-PML AML facilitates clinically valuable control of leukemia [12,18,27,28,29]. The therapeutic challenge lies in demonstrating that the various rationalizations for differentiation can be reconstituted therapeutically, with reduced toxicity in molecularly and genetically heterogeneous relapsed/refractory (r/r) non-PML AML.

Substituting the BCL-2 inhibitor venetoclax for a peroxisome proliferator-activated receptor alpha/gamma (PPARα/γ) agonist (pioglitazone) and an all-trans retinoic acid (ATRA) agonist (APA schedule) within the established azacitidine/venetoclax (Aza/Ven) schedule may promote the differentiation of blasts to neutrophils in r/r non-PML AML and induce leukemia cell death, as well as comprehensively ‘normalize’ myeloid hallmarks [18,25,30,31,32,33]. When applied at a reduced dose within the APA schedule, azacitidine acts as a facilitator for the activity of pioglitazone/ATRA [18]. Otherwise, neither of the nuclear receptor agonists would have any clinically relevant activity in r/r AML, while all-trans retinoic acid (ATRA) alone may dramatically promote differentiation in promyelocytic leukemia (PML) [25,26].

Successful differentiation induction in r/r non-PML AML reveals still pathophysiologically preserved and therapeutically actionable pathways if azacitidine may pave the way for triple transcriptional modulation by generating stress conditions in non-PML AML bone marrow tissue or extramedullary disease [18,28,34]. In vitro data show that the well-known monoactivity and differentiation-inducing capacity of azacitidine may be enhanced to restore phagocytic capability and to increase reactive oxygen species in non-PML AML neutrophils [18,25]. Even in cases of azacitidine failure, complete remission (CR) can be achieved, which highlights the novel synergistic activity profile of the APA drug cocktail [18,27]. CR may be established after just one cycle of APA therapy in patients with r/r non-PML AML [18].

This review summarizes the data from a phase I study of 10 patients with r/r non-PML AML who were treated with APA (azacitidine, pioglitazone and all-trans retinoic acid) together with published data on an additional seven cases of r/r non-PML AML treated with the APA protocol [18,27,28,29]. The treatment results for these 17 patients with r/r non-PML AML support the use of a drug combination that induces differentiation and leukemia cell death genome agnostically, even in patients with highly heterogeneous cytogenetic/molecular-genetic aberration patterns and complex pre-treatment conditions. For example, one patient had an early relapse following allogeneic hematopoietic stem cell transplantation (allo-HSCT) [29].

The data reveal that the differentiation associated with the improvement of immunosurveillance, as well as the attenuation of inflammation and the ‘normalization’ of leukemia metabolism by pioglitazone, may be followed by leukemia cell death and complete remission (CR), even in r/r non-PML AML, while synergistically targeting NOAs with three nuclear receptor agonists, the peroxisome proliferator-activated receptor (PPAR) α/γ agonist, the retinoid receptor RAR agonist all-trans retinoic acid (ATRA), and the epigenetic modulator, azacitidine [18,27,28,29,35,36].

## 2. APA Therapy

Patients selected for APA therapy were not eligible for dose-intensive therapy or allo-HSCT due to age or (treatment-related) comorbidity or due to insufficient induction response [18,27,28,29]. The time to progression (*n* = 16) or relapse (*n* = 1) was less than six months.

Between 2014 and 2020, a total of 17 patients with r/r non-PML AML received the APA schedule (azacitidine, pioglitazone and all-trans-retinoic acid). Ten of these patients were treated within a phase I trial of the German-Austrian AML Study Group (AMLSG) in Germany (the ViVA trial) [18], and seven consecutive patients also received APA and their cases were published in three papers due to their significant responses [27,28,29].

Patients in the AML ViVA trial were treated with an ATRA dose of 45 mg/m^2^/day from days 1 to 28, followed by 15 mg/m^2^ per os from day 29 onwards, in combination with subcutaneously administered azacitidine (75 mg fixed dose from days 1 to 7 of each 28-day treatment cycle) and pioglitazone (45 mg per os continuously from day 1). Non-study patients received APA with a fixed daily dose of ATRA (45 mg per os) continuously from day 1. Following APA induction, patients continued APA therapy for as long as was clinically appropriate, until AML relapsed or progressed. Cycle intervals beyond the first cycle were adapted to the time of hematopoietic regeneration.

## 3. Activity Profile of APA Therapy in Refractory or Relapsed Non-PML AML

Patients received a median of three cycles of the study treatment (range 1–14). The rapid onset of complete remission (CR) in r/r non-PML AML, even after one or two cycles, highlights the effectiveness of APA rescue therapy in r/r non-PML AML. Responding patients showed improvement or normalization of peripheral blood cell counts. However, early neutrophil recovery may be due to blast differentiation [27].

The manageable toxicity of APA enabled outpatient treatment for patients with CR and PR, including those aged 70 years and over (*n* = 4).

Preliminary data from a phase I trial (10 patients) and three case reports (7 patients) suggest APA may induce in r/r non-PML AML complete remission (CR), complete remission with residual thrombocytopenia (<100 G/L) or neutropenia (<1 G/L) (CRi), complete cytogenetic remission (CRc) or complete molecular remission (CRm). Ten of the seventeen patients (59%) who did not achieve CR in response to previous therapy experienced CR, CRi/c/m. APA maintained CR or PR for a long time (Table 1). As phase II or III data are still missing, the 59% CR rate must be interpreted as a preliminary observation from a heterogeneous and highly selective set of pooled publications. The CR rate is promising but currently does not represent a robust efficacy endpoint.

Two of the four patients aged over 70 remained in continuous complete remission (cCR) for over six months.

For patients with refractory non-PML AML, achieving eligibility for allo-HSCT is a high priority. The quality of remission prior to allo-HSCT is important for the long-term outcome [37]. Consolidation with allo-HSCT was facilitated by first-time CR with APA therapy in three of 16 patients with refractory non-PML AML.

The pathogenesis of de novo, secondary or treatment-related non-PML AML seems to have no significant impact on the response to APA therapy within small subgroups, as all three groups achieved CR, CRi/c/m: in de novo AML, three out of nine patients (33%); in secondary AML, one out of four patients (25%); and in treatment-related AML, three out of four patients (75%).

According to the 3-tiered venetoclax prognostic risk score (VEN-PRS), extramedullary disease is a poor prognostic parameter for aza/ven treatment [38]. One patient with complex aberrant cytogenetic involving chromosomes 5, 7, 8, 12, 13,18, 21, and 22 (Karyotype formula: 45,XX,der(5;7)(5pter->5p11::7q22->7q11::5p11->5q11::7q11->7pter)[4]; 45,XX, der(5;7)(5pter->5p11::7q22->7q11::5p11->5q11::7q11->7pter), der(12;18)t(12;18)(p13;q23)del(12)(p12p13)[2]; 45,XX,der(5;7)(5pter->5p11::7q22->7q11::5p11->5q11::7q11->7pter), +8,der(12;18)t(12;18)(p13;q23)del(12)(p12p13), ider(13) (q10)del(13)(q13q22), +21,+22[4]; 46,XX[3]; ISCN: nuc ish 7cen(D7Z1x2), 7q31(D7S486x1), 8cen(D8Z2x3), 12p13(3′ETV6x1,5′ETV6x2)(3′ETV6 con 5′ ETV6x1)[74/100]) achieved continuous CR of extramedullary non-PML AML in the skin. The multilineage dysplasia with ringsideroblasts (MDS-RS-MLD) remained stable [28].

Due to the small patient population, the observation that patients with complex karyotypes and no recurrent aberration (*n* = 7; 41%) responded poorly (100% no CR) may be accidental. Further studies should confirm this observation. At diagnosis, the following recurrent aberrations were routinely analyzed in the standard panel: MLLT3/MLL, CEBPA, PML/RARA, RUNX1, FLT3-ITD and FLT3-TKD, as well as NPM1.

In contrast, patients with complex karyotypes and additional molecular-genetic aberrations (*n* = 7, 41%) and those with molecular-genetic aberrations only (*n* = 2) responded with CRi/c/m (*n* = 6, 86%) and CR (*n* = 2, 100%), respectively. Considering molecular aberrations irrespective of complex aberrant karyotypes, eight out of nine patients (89%) achieved a CR.

Moreover, three high-risk patients with induction failure and a TP53 mutation responded: two achieved a CR, and one achieved a prolonged partial response (PR). As recently shown, this result was also achieved with decitabine plus ATRA [39]. According to the 3-tiered venetoclax prognostic risk score (VEN-PRS) for Aza/Ven treatment, CR in TP53-mutated AML is generally difficult to achieve, as is the case with NF1 and FLT3 mutations [40,41] (Table 1). FLT3 mutations commonly render cells resistant to aza/ven [39]. One patient with CMML, transformed into non-PML AML, had no aberration and could be reversed to CMML-2 with APA therapy [27].

Even a single intensive treatment regimen, including allo-HSCT, did not affect the likelihood of a positive response [29]. Patients who underwent one, two or three intensive treatment schedules achieved a complete response (CR), including a patient who had relapsed after allo-HSCT.

A total of seven patients died during the follow-up period. The median overall survival (mOS) in the ViVA trial was 131 days (i.e., 4.3 months).

## 4. Adverse Events on Patient Basis

The mean treatment duration for the 17 patients was 136 days (range 27–426 days), following a median follow-up period of 142 days. Major grade 3/4 events were likely due to baseline cytopenias caused by preceding treatment failure, AML progression, and toxicity from dose-intensive induction therapies (Table 2).

Expected hematological toxicities and infections are primarily attributable to the adverse starting conditions for APA therapy in advanced treatment lines. The bottom line of Table 3 compares the percentage of grade 3 or higher hematological toxicities in the ViVA Phase I trial with the mean percentage of hematological toxicities in the listed retrospectively analyzed Aza/Ven patients and the prospectively treated patients of the Aza/Ven Plus trials (additional drug(s) to Aza/Ven), as well as neutropenic fever and infection events (Table 3 and Table 4).

The incidence of grade 3/4 infection-associated adverse events (AEs) for the 17 summarized patients is similar to that published for the AML ViVA trial, which included 10 patients. Fever occurred in three patients (18%), lung infections in six patients (35%), urinary tract infections in four patients (24%), sepsis in one patient (6%), and other infections in two patients (12%) [18]. Hematological toxicities occurred in the additionally reported seven patients: five cases of grade 4 neutropenia, three cases of grade 3 thrombocytopenia and one case of grade 4 thrombocytopenia [27,28,29].

A large retrospective study of venetoclax-based HMA combinations in r/r AML shows grade ≥ 4 neutropenia in 100% of patients, grade ≥ 4 thrombocytopenia in 95.7%, and grade ≥ 4 febrile neutropenia in 45%. Compared to this toxicity data, APA treatment appears to be less myelosuppressive. Pioglitazone and ATRA do not increase hematotoxicity (Table 3) [45].

No additional non-hematological toxicities of grades 3 or 4 occurred in comparison to the ViVA study population in the 7 case reports. Non-hematologic toxicities grade 3 or 4 did not occur, which may be attributed to ATRA and pioglitazone, e.g., specifics on ATRA differentiation syndrome, pioglitazone effects on fluid retention, weight gain, or blood sugar decrease.

Serum glucose must principally be monitored in patients with diabetes mellitus when starting pioglitazone therapy. At baseline, the number of neutrophils in the peripheral blood and the percentage of blasts were very low in all 17 patients. Despite differentiation and release of neutrophils into the peripheral blood, no clinically relevant differentiation syndrome occurred. Principally, one should be prepared for a differentiation syndrome. Grade 1 clinically irrelevant fluid retention was observed in two patients due to pioglitazone.

The APA regimen was feasible and well tolerated in this early-phase study, even when considering the additional patients reported in case studies. It had a manageable toxicity profile that was distinct from that of intensive chemotherapy, and a tendency towards lower hematological toxicity, as observed in most retrospectively analyzed AZA/Ven studies or prospective AZA/Ven plus studies (see Table 3 and Table 4).

## 5. APA as Rescue Therapy for Azacitidine Failure

APA therapy successfully reversed the effects of azacitidine failure in three cases. Rescuing azacitidine failure with pioglitazone and ATRA illustrates the synergistic activity of the triple APA combination. Azacitidine failure generally indicates a poor prognosis for overall survival (OS), according to the three-tiered venetoclax prognostic risk score (VEN-PRS) [38]. Nevertheless, azacitidine paves the way for successful triple transcriptional modulation via receptor-triggered nuclear receptors, even though it was clinically ineffective as monotherapy [61].

Of the two patients with azacitidine failure, one with transformed chronic myelomonocytic leukemia (CMML) responded with CMML-2 after two cycles of APA therapy, while the other patient with an MLL-PTD mutation achieved a complete remission (CR) and received a successful allogeneic hematopoietic stem cell transplantation (allo-HSCT) as consolidation [27]. In a further patient, APA therapy rescued azacitidine failure following early relapse after allo-HSCT in a patient with high-risk AML. APA led to complete donor chimerism within one cycle [29].

Recently published results suggesting that PPARγ antagonists could be used to overcome decitabine/ven resistance contradict clinical results achieved with APA, as azacitidine resistance may be reversed by a PPARα/γ agonist (pioglitazone) when administered alongside APA [32]. The contradictory results might be linked to the missing coculturing of AML blasts with stroma cells in the experimental setting. However, data clinically achieved with APA are consistent with results from the same group showing that pioglitazone induces apoptosis in AML in vitro [62]. The specific impact of pioglitazone’s agonistic activity on PPARα cannot be determined [63,64,65].

**Figure 1:**
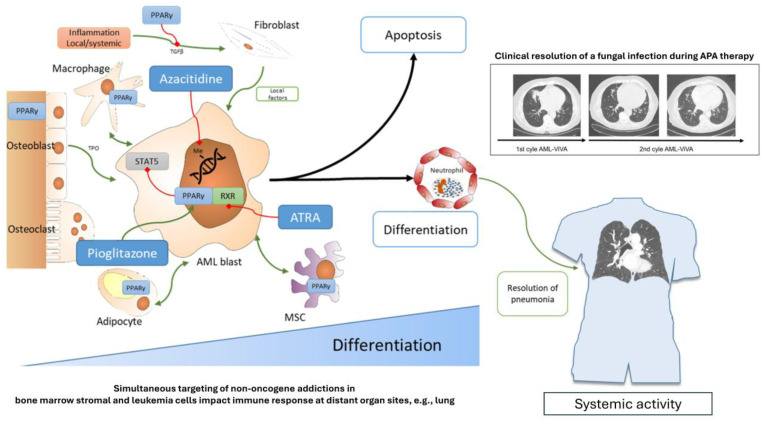
Simultaneously targeting non-oncogene addiction in both bone marrow stromal cells and leukemia cells using APA establishes myeloid hallmarks that impact the immune response in distant organs, such as the lungs. The figure depicts the effects of AML- APA therapy which leads to immunomodulation, anti-inflammation, modulation of leukemia metabolism and particularly to differentiation of leukemia blasts with phagocytosis activation in leukemic neutrophils, followed by leukemia cell death. AML blasts are embedded in the cellular bone marrow microenvironment and show complex stromal interplay. Successful pro-anakoinotic therapy in non-APL AML reveals that AML may be also considered as a non-leukemia-autonomous disease if non-oncogene addiction targets are concertedly addressed as therapeutic targets, here nuclear receptors. Approved but repurposed drugs may induce differentiation of AML blasts followed by clinically efficacious reconstitution of immunocompetence. Red blocked lines indicate inhibition; green arrows indicate support or agonistic behavior; PPARγ Peroxisome proliferator activated receptor γ; MSC mesenchymal stromal cell; TPO Thrombopoietin, ATRA all-trans retinoic acid; HMA hypomethylating agent.

**Figure 2:**
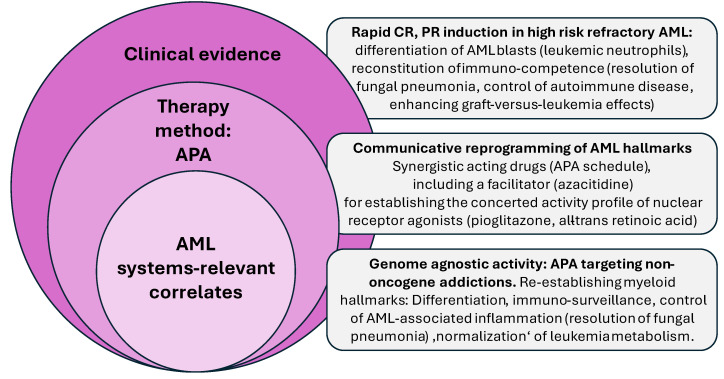
Therapy with APA (azacitidine, pioglitazone and all-trans retinoic acid): Remodelling non-oncogene addictions, biological and clinical results, and systems-relevant correlates in relapsed/refractory acute myeloid leukemia.

Contradictory preclinical and clinical data on the targeting of nuclear transcription factors may be resolved by considering the systems context of AML blasts and adjacent stroma cells, and by focusing in vitro and in clinical trials on drug combinations including at least one nuclear transcription factor agonist. For example, there have been positive clinical results for the combination of decitabine and ATRA in non-PML AML, and in the case of APA, even after preceding azacitidine failure [18,25,66,67] (Figure 1 and Figure 2).

Pioglitazone and/or ATRA alone are clinically ineffective in non-PML AML, although both drugs are physically interacting at their receptor sites [68]. Following the rescue of clinical azacitidine failure in r/r non-PML AML, the reciprocal biological activity of the two drug groups, pioglitazone/ATRA and azacitidine, becomes apparent [69]. Although azacitidine is clinically ineffective, it is found to be the decisive facilitator of pioglitazone/ATRA activity [18,25].

A recent experimental study shows that the resistance of AML blasts to pioglitazone may be mediated by stromal cells and can be overcome by an AXL inhibitor, bemcentinib [62]. These data support the simultaneous targeting of AML blasts and adjacent stroma cells to overcome resistance, as shown with the APA schedule in clinical practice. Dual versus triple exposure of AML blasts to APA drug components supports the clinically observed azacitidine rescue approach, as triple activity is a prerequisite for establishing cell differentiation and regaining the neutrophil activity profile in leukemic neutrophils [25].

The re-establishment of novel tissue homeostasis by reprogramming the communication between transcriptional networks maintained by tumor and adjacent stromal cells is called ‘anakoinosis’, a novel therapeutic principle [70,71]. Anakoinosis refers to the communicative reprogramming of cancer hallmarks, particularly those driven by neoplastic and adjacent stromal cells. Inducing differentiation, controlling inflammation, enhancing immunological surveillance, reprogramming tumor metabolism and tumor viability via NOA targets by tumor tissue editing approaches may be a way of controlling recurrent or refractory metastatic neoplasms, cancers, sarcomas and hematological neoplasms, up to the induction of CR and cCR [70,72]. Anakoinosis directly counteracts the reprogramming capabilities of oncogenes by selectively editing NOA circuitries that are communicatively cross-linked in tumor tissues and operated by oncogenic genomic events. Anakoinosis may enhance the accessibility of classic targeted therapies [73,74].

Clinical readouts following tumor tissue editing, e.g., genome-agnostic activity profiles, suggest that NOAs are organized into NOA-centered communicative systems, i.e., NOA circuitries, which can be accessed through tumor tissue editing. However, NOA circuitries are currently only inferred with functional modules and have not yet been defined quantitatively.

Azacitidine, like low-dose metronomic chemotherapy or MEK inhibitors, serves as the initiating drug for anakoinosis, implementing the prerequisites for the clinically efficacious application of triple transcriptional modulation, allowing differentiation induction and the re-establishment of immunosurveillance, as demonstrated both clinically and experimentally [18,25,72,75].

## 6. APA and Re-Establishment of Immuno Surveillance

Of the patients in the series who qualified for APA therapy as a last-resort treatment for r/r non-PML AML, two suffered from additional autoimmune diseases: one had psoriasis involving the large joints, and the other had systemic lupus erythematosus [76,77]. However, pre-treatment with cytotoxic agents did not sufficiently control the autoimmune diseases. Both diseases were finally controlled after one cycle of APA therapy, revealing APA’s strong immunoregulatory activity profile, as has been demonstrated for APA-like therapies, including metronomic low-dose chemotherapy, in non-hematological neoplasias [72,76,78].

Consistent with this is the finding that restored immunocompetence can treat severe fungal pneumonia acquired during intensive induction therapy and induction failure (Figure 1). The fungal pneumonia was resolved after two cycles of APA therapy, after which the patient qualified for allo-HSCT, which was successfully performed [18].

Regaining immune surveillance during APA therapy may be due to the immunoregulatory effects of the drugs themselves, as well as the differentiation of blasts into neutrophils being associated with the restoration of neutrophil functions [25,79,80]. This has been demonstrated experimentally, with the reacquisition of phagocytic activity and increased reactive oxygen species (ROS). These are both prerequisites for processing microbial infections [18,25].

Targeting NRF2 with pioglitazone and attenuating the oxidative stress and stress conditions induced by azacitidine may ultimately enhance immune surveillance [20,65,81,82,83]. Experimental data show that T-cell-specific response to breast cancer is possible in the combination of metronomic chemotherapy plus pioglitazone and also following DNA methyltransferase inhibition [78,80].

Achieving complete donor chimerism with APA, but not azacitidine, reveals a synergistic immunological effect of APA with the graft-versus-leukemia (GvL) reaction. Differentiation of blasts may again play an important role here, as the overexpression of antigens such as proteinase 3 and other azurophil granule proteins may serve as targets for allogeneic and autologous T-cell responses [84,85,86]. Azacitidine-associated DNA methyltransferase inhibition may further enhance T-cell responses by inducing interferon and upregulating major histocompatibility class I (MHC-I) genes [80,87].

## 7. Differential AML Hallmarks Targeted with Pioglitazone, ATRA

In addition to clinical and experimental observations regarding the re-establishment of myeloid hallmarks in AML blasts and the differentiation and reconstitution of immune surveillance, APA modulates the metabolism of leukemia cells (e.g., leukemic neutrophils) and the inflammation that promotes AML, as well as microbial inflammation at a distant site (e.g., resolution of fungal pneumonia) [18,25,38]. The combination of pioglitazone and ATRA targets both AML blasts and stromal cells, reestablishing hallmarks that are therapeutically reconstituted via concerted regulatory activity in AML blasts and adjacent stroma [18,22,25,68]. Distant inflammation, manifested as a fungal infection, may benefit from the therapeutic re-establishment of myeloid hallmarks.

Inflammation is considered to be at the forefront of AML pathogenesis, as has recently been demonstrated [38,88]. A blood-based leukemia inflammatory risk score (LIRS) is a significantly better predictor than the current European LeukemiaNet 2022 risk model, which considers genetic, molecular-genetic and clinical risk factors [19]. LIRS also serves as an independent prognostic factor for overall survival based on both known clinical risk profiles and inflammation-based markers in serum [19]. Thus, inflammation control by pioglitazone for AML control resumes the originally approved activity profile in insulin-resistant diabetes mellitus, inflammation control [35,88,89,90]. Moreover, attenuation of pro-inflammatory drivers of AML pathogenesis might explain alone genome agnostic activity of APA. As recently shown, differential APA-like schedules designed for multiple r/r neoplasias reveal the correlation of serum C-reactive protein response and neoplasia control [19,72].

The Leukemia Inflammatory Risk Score (LIRS) uses blood-based pro-inflammatory biomarkers to predict AML survival [19]. These markers provide a key rationale for substantial anti-inflammatory therapy strategies, e.g., pioglitazone for AML control [18,38]. Interestingly, the LIRS remains relevant regardless of the intensity of previous treatment [19].

APA-like schedules have principally demonstrated potent anti-inflammatory effects in various neoplasias, cancers, including hematological neoplasias, when treated with metronomic low-dose chemotherapy in conjunction with additional transcriptional modulators. These schedules can control relapsed/refractory neoplasms up to the induction of continuous CR (cCR) [72,91].

An excellent example of how pioglitazone attacks leukemia stem cells in chronic myeloid leukemia (CML) has been demonstrated by Prost et al. through its interaction with imatinib in patients whose imatinib molecular response is failing [35,92]. Once again, the addition of pioglitazone or rosiglitazone can revitalize the activity profile of monotherapies that have failed, such as imatinib, azacitidine, MEK inhibitors and gefitinib (an EGFR TKI) [18,27,28,92,93,94].

Broadly speaking, a variety of approaches to therapy that induce anakoinosis with APA-like therapies may focus on establishing distinct disease-relevant hallmarks that are lost during neoplastic transformation. These approaches can be used to control recurrent or refractory neoplasms (hematological neoplasms, sarcomas and cancers), and presumably r/r non-PML AML as well [35,72,73].

Experimental designs considered the therapeutic use of PPARγ agonists/antagonists. Resistance to decitabine/ven in AML may be induced by the upregulation of fatty acid metabolism, which is thought to be counter-regulated by PPARγ antagonists [32]. The antileukemic activity of selenium may be mediated by activation of PPARγ via the agonist prostaglandin J2. [95]

Drugs that target oncogenic aberrations in AML might enhance the biomodulatory effects of therapeutic approaches such as APA. Clinical trials have included the FLT3 inhibitor gilteritinib as an adjunct to Aza/Ven. In FLT3-positive relapsed/refractory AML, the CR/CRi rate was 27%, but impressive toxicity remains a striking problem [16]. ATRA may increase cell death induced by quizartinib [96].

## 8. APA and AML Microenvironment

The resolution of extensive leukemic skin infiltration in a patient with treatment-related MDS-RS-MLD, but no increased blast count in the bone marrow, impressively demonstrates the impact of environmental context (bone marrow versus skin) on leukemia growth control with APA therapy. The MDS-RS-MLD transformed into AML after 210 days of APA treatment, during which there were no skin relapses [28]. Despite repetitive therapies for bone marrow AML, the leukemic skin infiltration did not recur. These observations highlight the importance of simultaneously targeting AML blasts and adjacent stromal cells to effectively control AML.

Myeloid hallmarks may be reinstated by APA-mediated tissue reprogramming of AML. Myeloid hallmarks are collectively constituted via targeting of AML blasts and adjacent stromal cells [83,88]. The targeted nuclear receptors are typically distributed throughout the stroma and leukemia cells. PPARγ expression may change depending on the context [35,62].

The adaptation of the transcriptionally active drug cocktail for use in r/r neoplasms may be genome-agnostic, as demonstrated in r/r multisystem Langerhans cell histiocytosis (mLCH) and r/r renal clear cell carcinoma (RCCC). In patients with r/r mLCH who failed to respond to treatment with pioglitazone and dexamethasone, substitution of the transcriptionally active drug cocktail with interferon-α and pioglitazone—a schedule that had previously been successfully tested for r/r renal clear cell carcinoma—resulted in cCR. Each transcriptional cocktail was combined with metronomic low-dose chemotherapy [73,97,98,99].

As with the combination of a PPARγ agonist and interferon-α for controlling r/r RCCC/mLCH, the combination of a PPARγ agonist and a RAR agonist (ATRA) can also be successfully used in a genome-agnostic manner for treating r/r non-PML AML or muscle-invasive bladder cancer (in a mouse model) [72,74]. Facilitators of efficaciously combined transcriptional intervention include metronomic low-dose chemotherapy, azacitidine and MEK inhibitors, revealing the potential for further systems pharmacological exploitation [74].

## 9. APA Therapy Predominantly Targets Non-Oncogene Addictions

Clinically used azacitidine doses predominantly exert their activity through hypomethylating effects [14,100,101]. As AML blasts depend heavily on controlling DNA methylation, the epigenetic modifier azacitidine is a key inhibitor of the methylation process, notably in a non-oncogene-addicted manner [100,101]. Consequently, azacitidine neutralizes the suppression of tumor suppressor genes in parallel with pioglitazone [101,102]. RNA-mediated cytotoxic effects are not the main mechanisms of azacitidine action at clinically applicable doses [101].

Response to or primary resistance to azacitidine in non-PML AML cannot be predicted from specific mutational signatures, as azacitidine activity is not predominantly disruptive via cytotoxicity, but may reprogram AML cell fate by establishing myeloid hallmarks genome agnostically, such as differentiation [34,103].

Azacitidine makes full use of the plasticity of AML tissues, particularly by inducing novel stress responses in neoplastic cells. For example, it targets stress dependency on the antiapoptotic BCL-2 family proteins BCL-XL, BCL-2 and MCL-1 [104,105]. The clinical efficacy of azacitidine in combination with the BCL-2 inhibitor venetoclax indicates the therapeutic importance of the epigenetic modulation of NOAs in the treatment of AML.

Compared to approved cytotoxic or Aza/Ven therapies, APA is a less toxic, synergistic, bioregulatory combination therapy [18,45]. The rationale behind APA is to induce AML cell death by re-establishing myeloid hallmarks in non-PML AML blasts, probably also in cases that are resistant to decitabine/ven [32,106].

Azacitidine was administered at a lower dose than the approved one in the APA schedule. In the APA schedule, azacitidine predominantly serves as a facilitator for the re-establishment of AML plasticity (Figure 1). Unlike azacitidine in the Aza/Ven schedule, which has predominant cytotoxic effects, azacitidine in the APA schedule acts as a ‘facilitator’ to recover bioregulatory processes [18,25,107].

In non-PML AML, clinically non-functional transcriptional regulators such as pioglitazone and ATRA cannot unlock AML tissue plasticity independently of azacitidine’s epigenetic regulation [18,25,29]. Preclinical data reveal a synergistic activity profile of pioglitazone and ATRA in a variety of neoplasias [25,107,108,109].

Inducing stress dependencies in the bone marrow tissue of AML patients through APA therapy leads to obvious genome-agnostic failure of the tissue to adapt to APA-induced stress, resulting in the development of resistance mechanisms [110]. Conversely, a targeted and coordinated reorganization of myeloid hallmarks occurs in AML blasts through the reciprocal activity of APA drugs. This enables APA to overcome AML addictions and establish the ability to differentiate into neutrophils under disease conditions [18,25]. Leukemia plasticity, which is often discussed as a prerequisite for the development of resistance, is now therapeutically exploited by involving the AML-silenced but still organizable networks of NOAs and NOA circuitries that contribute to differentiation in AML [111] (Figure 2).

In summary, APA therapy promotes the plasticity of non-PML AML blasts by reprogramming the NOA networks in non-PML AML to reintegrate ‘normalized’ myeloid hallmarks.

However, there is variability in the rationalizations of the processes that guide differentiation and the restoration of immune competence, as demonstrated by the poor responses observed, particularly in AML patients with genetic aberrations only. APA therapy successfully covers a wide range of chromosomal and molecular genetic aberrations due to the frequently occurring, uniquely organized NOA networks that facilitate the differentiation of blasts and the re-establishment of immune competence [80,85,112,113].

This principally demonstrates that NOAs provide a significant means of achieving a clinically meaningful response in r/r non-PML AML [18,85]. The huge heterogeneity of chromosomal and molecular genetic aberrations in non-PML AML contrasts with the limited diversity of rationalizations that facilitate myeloid differentiation and the reconstitution of immune competence. Due to the genome-agnostic activity of the APA schedule in mediating differentiation induction, a limited number of schedules, such as APA or APA-like schedules, seem necessary to cover differentiation in AML blasts, despite the significant genetic heterogeneity.

## 10. APA, a Genome Agnostic Therapy

Descriptive statistics show that only one subgroup of refractory non-PML AMLs responds poorly to APA: those with chromosomal aberrations only. Despite the significant heterogeneity of molecular and genetic aberrations, the unfavorable prognostic features of the 16 refractory patients and one relapsed did not preclude the possibility of an APA response with complete remission (CR), complete remission with incomplete blood count recovery (CRi), complete remission with partial blood count recovery (CRi) or complete remission with minor blood count recovery (CRm). CRs occurred in elderly patients (≥70 years), in de novo, secondary or treatment-related AML, and in patients with a high number of pre-treatments. This occurred independently of molecular-genetic aberrations, plus/minus complex aberrant karyotypes.

The wide range of genetic aberrations in AML blasts within the APA cohort, and the unique, therapeutically successful targeting of growth-promoting AML hallmarks with APA, reveal that APA activity is genome-agnostic (Table 1, Figure 2).

Novel targeted therapies that induce differentiation in AML blasts are predominantly active in the presence of a single recurrent mutation; therefore, they are not generally applicable. Isocitrate dehydrogenase 1 and 2 inhibitors, menin inhibitors and FMS-like tyrosine kinase 3 inhibitors can cause lysis syndromes similar to those seen with all-trans retinoic acid (ATRA) therapy for PML [114].

These recurrent mutations may be detected in 12–25% of non-PML AML cases and comprise key genetic drivers of AML (e.g., KMT2A). Their occurrence may also increase with age [115,116]. Targeting these mutations therapeutically to unlock AML differentiation may elicit a response in patients who have not responded to previous AML therapies. However, the APA cohort studied comprises a sizable proportion of genetically heterogeneous AML patients who may benefit from APA therapy inducing anakoinosis. The comparison of APA-mediated differentiation versus differentiation induction by inhibitors of recurrent mutations reveals that AML blasts may provide different rationalizations for initiating differentiation (Figure 2). In contrast to therapies targeting recurrent mutations, APA therapy is multi-level and restores a pattern of stroma-linked hallmarks, differentiation, immune response and inflammation control and AML metabolism. This has also been demonstrated in a variety of r/r neoplasias [69,72,117].

## 11. Second-Line Aza/Ven Versus APA in r/r Non-PML AML

Selecting salvage therapies for patients with relapsed or refractory AML remains challenging and controversial, as response rates are poor [12,30,118,119].

Published second-line response and overall survival (OS) rates to Aza/Ven are compared to those achieved with APA. As Aza/Ven is frequently used in the first line of treatment for elderly or medically unfit patients, it is often not considered as a second-line treatment, so alternative approaches are welcome [120].

Importantly, Aza/Ven has changed the prognostic parameters for first- and second-line AML treatment compared to classic intensive induction therapies [38]. Aza/Ven responses demonstrate a novel therapeutic approach to AML compared to chemotherapy [121].

A large retrospective study developed a three-tiered venetoclax prognostic risk score (VEN-PRS) for azacitidine/venetoclax-treated r/r AMLs. According to the novel VEN-PRS scoring system, the APA patient cohort comprises patients at adverse risk, as indicated in Table 1. The median overall survival (OS) for the ViVA trial cohort (*n* = 10 patients) was 131 days (i.e., 4.3 months), reflecting the OS of the adverse patient population with a VEN-PRS of 4.6 months (1.7–8.2 months) (Table 3) [38].

The APA patient cohort (*n* = 17) comprises patients at adverse risk, 10 of whom (59%) have mutations in TP53 (*n* = 3), NF1 (*n* = 1) and FLT3 (*n* = 2) (Table 1). Furthermore, three patients experienced failure of azacitidine pretreatment, and one patient had extramedullary AML, according to the adverse risk parameters included in the VEN-PRS. Additionally, the APA cohort was characterized by multiple myelodysplasia-related gene mutations, which are associated with poor outcomes based on classic intensive AML therapies. Overall survival (OS) was significantly shorter for patients with RUNX1 (*n* = 2), SF3B1 (*n* = 0) and U2AF1 (*n* = 1) mutations compared to patients in the ELN intermediate risk group [39,122].

In summary, 11 patients (65%) harbor dismal prognostic genetic parameters. According to the 2022 ELN genetic risk category, all APA patients who were treated were at an adverse risk [123].

Table 3 lists available cohorts of patients with r/r non-PML AML treated with Aza/Ven (retrospectively analyzed data) and available median overall survival rates [18,38,42,43,44,46,47,48,49,50,51,52,124]. The heterogeneous median overall survival rates indicate highly heterogeneous patient populations treated with Aza/Ven in the r/r stage due to pre-treatment, cytogenetic/molecular-genetic and clinical risk factors.

Prospective phase II/III trials with accompanying detailed diagnostics are necessary to evaluate the impact of Aza/Ven in the r/r stage. Further large-scale APA trials are necessary to fully demonstrate the impact of APA on survival.

Although most trials including Aza/Ven plus additional drug(s) were performed prospectively, the clinical impact of extending the azacitidine/venetoclax schedule with chemotherapy or targeted therapy cannot yet be fully assessed. Once again, the treated populations are highly heterogeneous [53,54,55,56,57,58,59,60].

One trial including trametinib should be highlighted (see Table 4), as the trial design exemplifies the importance of tumor tissue editing with pioglitazone/ATRA in non-PML-AML. Combining trametinib with rosiglitazone and ATRA induces objective response in a mouse model of muscle-invasive bladder cancer; however, Aza/Ven plus trametinib in AML does not [58,74]. This example demonstrates that communicative reprogramming using distinct combinations of transcriptional modulators can be achieved across different tumor types, regardless of the genomic aberrations. Simply adding trametinib to Aza/Ven fails to induce a significant response in r/r AML with RAS mutations [58].

The change in prognostic parameters with Aza/Ven therapy versus chemotherapy indicates a significantly different pathophysiological approach to AML blasts compared to dose-intensive chemotherapy. APA uses one cytotoxic agent (azacitidine at a reduced dose of 45 mg per day from day 1 to day 7 of a 28-day treatment cycle) and two non-hematotoxic transcriptional agents, instead of 400 mg of venetoclax per day from day 1 to day 7 of a 28-day treatment cycle.

The introduction of the APA schedule indicates more than just a substitution of a cytotoxic therapy element, venetoclax, as targeted therapy. The triple combination of APA comprehensively reveals molecular and biological changes in the organization of NOAs as novel therapeutic principles through the synergistic and biomodulatory interaction of the APA drugs. Hidden myeloid functions in AML blasts that guide AML cell death can be re-established, even in patients who have been pre-treated with azacitidine [18,27,28,29,45,125,126]. Therefore, azacitidine-induced tRNA methylation, which may redirect the translational response to stress, does not appear to be sufficient for coordinating myeloid differentiation with clinical significance [34].

As neutropenic infections are a major cause of mortality in AML patients, they should be avoided through reducing treatment-related hematotoxicity and providing supportive care [11]. Until now, the restoration of myeloid cell functions in AML blasts and the enhancement of immunosurveillance by the APA schedule appeared to be incompatible with the induction of AML cell death. Initially observed APA hematotoxicity is primarily related to the AML bone marrow infiltrate and preceding intensive induction treatment toxicity and APA-related non-hematological toxicities are manageable (Table 1). Beyond cycles one or two, all subsequent cycles can be administered in an outpatient setting.

## 12. No Long-Term Continuous Complete Remission Without Allo-HSCT Consolidation

Long-term observation of patients treated with aza/ven in the first line, with a median follow-up of 43.2 months, shows a steadily declining survival curve, indicating that a cure may not be achievable for most patients, except those with favorable risk parameters such as NPM1 mutations [127,128,129].

The same continuous decline in survival curves can be expected for patients treated with APA if they do not qualify for allo-HSCT after treatment. Interestingly, long-term AML control (426 days) can be achieved without reaching CR. A cure for r/r non-PLM AML was possible in cases of extramedullary leukemic manifestation (skin) or via consolidation with allo-HSCT (*n* = 3). The patient who received allo-HSCT after APA treatment relapsed before the start of the seventh APA cycle.

APA facilitates CR and, consequently, successful consolidation with allo-HSCT and long-term maintenance of CR or PR, even in patients with TP53 mutations and other poor-risk mutations, as indicated in Table 1.

The advantages of APA therapy are compelling, despite being based on a small cohort. These include:

APA activity after azacitidine failure;

Rescue of poor-risk cytogenetics and molecular genetics with APA;

Manageable toxicity of APA in consecutive cycles if CR or PR has been achieved;

Generation of prerequisites for allo-HSCT in patients who are primarily ineligible;

Rescue of allo-HSCT failure;

Facilitation of long-term stabilization of CR and PR;

Last but not least, APA therapy can address the needs of elderly and frail patients.

## 13. Discussion

In the case of r/r AML, the introduction of low-dose cytotoxic facilitators such as azacitidine is a prerequisite for the successful implementation of targeted transcription modulation in AML blasts and adjacent tissue, and for the concerted reprogramming of AML hallmarks. As demonstrated in clinical trials, these facilitators also include low-dose metronomic chemotherapy and decitabine [130,131]. As demonstrated in numerous clinical trials, facilitators that enable the targeted modulation of tissue-specific cancer hallmarks can induce stress responses in neoplastic tissue [82]. This creates the prerequisites for the reorganization of neoplasia-associated hallmarks, which is of particular importance in terms of therapy of r/r neoplasias [73,82,132,133,134]. As demonstrated in the context of APA-like schedules, metronomic low-dose chemotherapy can facilitate genome- and even neoplasia-agnostic schedules [72].

APA omits major non-hematological side effects in normal, non-addicted tissues, thereby highlighting the specificity of the pro-anakoinotic process. The induction of CR in r/r non-PML AML in a genome-agnostic manner reveals the therapeutic accessibility of a large, well-organized set of NOA targets, i.e., NOA circuitries, for achieving pivotal, therapeutically relevant leukemia plasticity. Reprogramming of NOA circuitries can be done by reactivating hidden myeloid hallmarks in AML tissue [18,130].

As clinically demonstrated, pioglitazone- and ATRA-redirected AML tissue-triggered hallmarks comprise differentiation and enhanced immuno surveillance, as well as inflammation control and ‘normalized’ metabolism. As AML inflammation scores demonstrate high predictive value for outcome, controlling inflammation with pioglitazone, as demonstrated for other r/r neoplasias treated with APA-like schedules, may also be pivotal in controlling r/r non-PML AML with APA-like schedules [38,88].

Considering the adverse side effects of current chemotherapies, most targeted therapies or the Aza/Ven schedule for controlling non-PML AML, differentiation induction combined with the regain of immune competence and inflammation control with APA are contradictory treatment experiences compared with chemotherapy [135].

Phase II/III trials on APA are necessary to establish the APA schedule clinically. However, the presented APA data exemplifies the novel, genome-agnostic therapy principle and the new, systems-relevant correlates that lead to AML cell death (Figure 2). Further studies must elucidate the precise molecular mechanisms of APA therapy within a systems context. Meanwhile, pre-clinical data on pioglitazone in AML are continuously adding to our understanding of its activity in neoplasias [22,35,82,136].

The fact that APA treatment has made previously ineligible patients eligible for allo-HSCT is remarkable, given their pre-APA infection history and the response quality induced by APA [137]. Thus, APA made successful allo-HSCT possible and facilitated cures in high-risk patients, including the elderly.

Targeting NOA networks in a concerted manner using APA-like therapy strategies enables specific adaptation to the pathophysiological requirements of refractory neoplasias and offers novel therapeutic development possibilities [72]. (1) A phase II/III trial should be established to confirm the promising APA results, particularly in light of the novel data showing inflammation-triggered AML pathogenesis [19]. On the other hand, extended experience from clinical trials on inflammation control with pioglitazone in relapsed/refractory neoplasms treated with APA-like schedules reveals the therapeutic relevance of attenuating neoplasia-associated inflammation in order to achieve continuous CR [19,35,72,91]. (2) Furthermore, the differential combined targeting of receptor-triggered transcription factors enables the reprogramming of the pathogenetically most relevant AML hallmarks, with the aim of establishing the most effective genome-agnostic leukemia control. This is achieved by therapeutically coordinating NOA networks, i.e., anakoinosis, which promote the restoration of myeloid hallmarks lost during malignant transformation to AML [18,25,27,28,29]. (3) Additionally, oncogene-addicted targets may be therapeutically addressed in the context of APA-like schedules. Even myelotoxic drugs can be added due to APA’s manageable toxicity profile, distinct from intensive chemotherapy, particularly after rapid CR or PR induction [13,72]. (4) The diverse facilitators of effective combined receptor-triggered transcriptional modulation for the control of recurrent and refractory neoplasms reveal novel systems pharmacological activity profiles in a genome- and tumor-agnostic manner [18,72,74]. (5) Proteomic platforms and tissue mass spectrometry imaging may enable the testing of novel pharmacological approaches for AML ‘normalization’ in vitro, as indicated by the therapeutic establishment of myeloid hallmarks [13,72,73,136,138,139].

## Figures and Tables

**Table 1:** AML diagnostics/history of pre-treatment and response to APA therapy.

Variable	Best Response	N Cycles/No. of Days on APA Treatment	N = 17
**Sex, N (%)**			17 (100)
Male	-	-	11 (65)
Female	-	-	6 (35)
Age in years, median (range)	-	-	66 (55–76)
Age >= 70 years	3x CR, 1x SD (1x tAML, 3x de novo)	-	4 (70–76)
**Type of AML, N (%)**			
de novo AML	3x CR, 1x PR, 4x SD, 1x NA	CR 10/350, 4/240, 4/140; 5/175; PR 14/490; SD 1 to 3; NA-	**9 (52)**
secondary AML	2x SD, 1x PR, 1x CR	SD 2x 1/30; PR 3/110; CR 5/210	**4 (24)**
treatment-related AML	2x CR, 1x Cri, 1x CR skin and SD of MDS-RS-MLD	2 to 6/70 to 210	**4 (24)**
**ECOG performance score, N (%)**			
0	-	-	3 (18)
1	-	-	12 (70)
2	-	-	2 (12)
**Cytogenetics at baseline, N (%)**			
complex karyotype (+/− molecular genetic aberrations)	SD, PR, CR	**-**	**14 (82)**
§ Complex karyotype involving chromosomes 5, 7, 8, 12, 13, 18, 21, and 22	1 CR skin	6/210	1 (6)
del(9), del(3) *(each with molecular genetic aberrations)*	2 Cri	4/140; 5/210	2 (12)
No complex karyotype *(MLL-PTD, FLT3-TKD* mutation, *1x no aberration: AML from CMML)*	2x CR, 1x PR	2/74; 5/175; 3/110	**3 (17)**
**Molecular genetics (mutations) at baseline, N (%)**			
None * but complex karyotype	*1x PD*, *5x SD*, *1xPR*	*1 to 4/30 to 140*	7 (40)
No aberration (secondary AML from CMML with 25% ** blasts)	PR (CMML-2)	3/110	1 (6)
*TP53*	PR	14/ 426	1 (6)
*TP53*, *ASXL1*	CR	10/ 350	1 (6)
*TP53*, *NF1*	CR ***	2/70	1 (6)
*ASXL1*, *IDH2*, *SF3B1*, *U2AF1*	CR ***	2/75	1 (6)
*t(8;21)*, *monosomy Y*, *RUNX1-RUNX1T1*, *ETV6*	Cri/c/m	4/240	1 (6)
del(3), *MLL*-PTD, *FLT3*-ITD, *RUNX* mutation	Cri/c/m	5/210	1 (6)
del(9), trisomy 4, *MLL-PTD*	CR, CRc/m	4/140	1 (6)
*MLL-PTD*	Cri ***	2/74	1 (6)
*FLT3-TKD* mutation	Complete chimerism after 1cycle	5/175	1 (6)
**ELN 2022 genetic risk category, N (%)**			
adverse	-	-	17 (100)
**N and type of pretreatment, N (%)**			
**1 cycle**			**9 (53)**
cytarabine/daunorubicin (DA)	CR, SD	-	7 (41)
thioguanine/cytarabine/daunorubicin (TAD)	NA, SD	-	1 (6)
Azacitidine (AZA)	SD	-	1 (6)
**2 cycles**			**6 (35)**
cytarabine/daunorubicin (DA); mitoxantrone/cytarabine (HAM)	CR	-	3 (17)
thioguanine/cytarabine/daunorubicin (TAD); mitoxantrone/cytarabine (HAM)	PD	-	1 (6)
Azacitidine (AZA); cytarabine/daunorubicin (DA)	CRi	-	2 (12)
**3 cycles**			**2 (12)**
cytarabine/daunorubicin (DA); mitoxantrone/cytarabine (HAM); mitoxantrone/etoposide/m-amsacrine/cytarabine (MAMAC)	CR	-	1 (6)
cytarabine/daunorubicin (DA); mitoxantrone/cytarabine (HAM)/midostaurin; **allo-HSCT** ***, azacitidine	CR	-	1 (6)

* Analyzed in standard panel: *MLLT3/MLL*, *CEBPA*, *PML/RARA*, *RUNX1*, *FLT3-ITD*, *FLT3-TKD*, *NPM1*. § Patient with bone marrow and skin involvement. MDS-RS-MLD: Multilineage dysplasia and ringsideroblasts. ** BM cellularity > 90%. AML: acute myeloid leukemia; CMML: chronic myelocytic leukemia; ECOG: Eastern Cooperative Oncology Group; ELN: European LeukemiaNet. DA: Daunorubicin (60 mg/m^2^ on days 3–5), cytarabine (100 mg/m^2^ on days 1–7); TAD: daunorubicin, cytarabine and oral thioguanine 100 mg/m^2^ q 12 h on days 3 to 9. HAM: high-dose cytarabine (1 g/m^2^ per q12h on days 1–3), mitoxantrone (10 mg/m^2^ on days 3–5); MAMAC: high-dose cytarabine (1 g/m^2^ per q12h on days 1–5), amsacrine (100 mg/m^2^ on days 1–5); AZA: 5-azacytidine; *** allo-HSCT: allogeneic hematopoietic stem cell transplantation,; NA not available. Red: risk-factors for inferior OS, Green: favorable risk factor (3-tiered venetoclax prognostic risk score (VEN-PRS)).

**Table 2:** Hematologic parameters before APA treatment.

Variable	Readout
Hemoglobin g/dL, median (range)	8.8 (6.3–10.6)
Platelets ×10^9^/L, median (range)	48.3 (5–175)
White blood count ×10^9^/L, median (range)	0.93 (0.33–4.8)
Neutrophils ×10^9^/L, median (range)	0.19 (0–2.7)
Bone marrow blasts %, median (range)	58 (10–90)
Peripheral blood blasts %, median (range)	0 (0–26)

**Table 3:** Studies on azacitidine/venetoclax (Aza/Ven) in relapsed/refractory non-PML AML compared to APA and Aza/Ven +/− additional drugs: median overall survival and available toxicity data (studies marked with bold text in Table 3 and Table 4 are summarized in the bottom line of Table 3 for comparison of the available adverse event data).

	Aza/Ven	Clinical Outcome	Adverse Events >= Grade 3					
	Relapsed/Refractory AML No of Patients Retro-/Prospective Study	Median Overall Survival (Months) 4.0 to 17 Months	Anemia %	Thrombo-Cytopenia %	Neutropenia %	Neutropenic Fever %	Infection %	Literature
Garciaz S, 2022	39 retrospective	5.9	n	n	n	n	n	[42]
**Petit C, 2024**	**35 retrospective**	**12.8**	**n**	**n**	**n**	**51.4**	**n**	[43]
Stahl M, 2021	35 retrospective	25	n	n	n	n	n	[44]
**Piccini M, 2021**	**47 retrospective**	**10.7**	**n**	**95.7**	**100**	**45**	**n**	[45]
Angotzi F, 2024	37 retrospective	11.9	n	n	n	n	n	[46]
**Aktimur SH, 2020**	**30 retrospective**	**7.0**	**-**	**100**	**83.3**	**70**	**n**	[47]
Lou Y, 2020	48 retrospective	9.6	**n**	n	n	n	n	[48]
Labrador, 2022	30 retrospective	4.0	n	n	n	n	n	[49]
**Bouligny, 2022**	**22 retrospective**	**17.0**	**81**	**85.7**	**95.2**	**38.1**	**38.1**	[50]
**Xu X, 2023**	**31 retrospective**	**-**	**87.1**	**83.9**	**90.3**	**n**	**n**	[51]
Morsia E, 2020	42 retrospective	5.0 (17 vs. 3 months without CR/CRi, *p* < 0.001	n	n	n	n	n	[52]
**Shahswar R, 2025**	**258 retrospective** **Molecular cohort: n = 174**							
	**adverse** (n = 53)	**4.6**						
		(Risk factors for inferior survival included the presence of extramedullary disease, HMA pretreatment and mutations in NF1, PTPN11, FLT3, and TP53)	**-**	**-**	**-**	**-**	**-**	[38]
	**Intermediate** (n = 75)	**7.5** (*p* < 0.001)						
	**Azacitidine,** **Pioglitazone and ATRA**							
**Heudobler D, 2023**	**10** prospective	**4.3** Risk factors (Table 1): extramedullary disease, NF1, FLT3, TP53, preceding azacitidine failure, t-AML	**60**	**40**	**50**	**20**	**40**	[18]
	**Aza/Ven +/− additional drugs**							
**Mean values of studies** **(Table 3 and Table 4, bold text)**	retro-/prospective	**-**	**77.7** (6 studies)	**98.7** (8 studies)	**90.8** (8 studies)	**49.2** (7 studies)	**38.6** (5 studies)	-

**Table 4:** Studies on azacitidine/venetoclax (Aza/Ven) +/− additional drugs in relapsed/refractory non-PML AMLs: median overall survival and available toxicity data (studies marked with bold text are summarized in the bottom line of Table 3).

	Aza/Ven Plus	Phase	Clinical Outcome	Adverse Events >= Grade 3					
	Relapsed/Refractory	Number of Patients	Median Overall Survival (Months)	Anemia %	Thrombo-Cytopenia %	Neutropenia %	Neutropenic Fever %	Infection %	Literature
**Yu S, 2024**	**Homoharringtoine, sorafenib**	**51** **Phase II**	**18.1**	**68.6**	**80.4**	**92.2**	**56.9**	**37.2**	[53]
**Jin H, 2023**	**Homoharringtoine**	**96** **Phase II**	**22.1**	**66.7**	**75**	**82.3**	**37.5**	**38.5**	[54]
**Yu G, 2025**	**Homoharringtoine**	**172** **Phase II**	**n**	**66.3**	**73.3**	**83.1**	**45.9**	**36**	[55]
Short, 2022	Gemtuzumab ozogamicin	21 Phase IB/II	7.6	n	n	n	28.6	30.1	[56]
Zhao P, 2022	Donor lymphocyte infusion (DLI)	26 retrospective	9.5	53.8	100	100	n	n	[57]
Desikan SP, 2022	Trametinib (RAS mutated)	15 Phase II	2.4	n	n	n	n	n	[58]
**You L, 2024**	**Cytarabine**	**30** **Phase II**	**Not reached** **Median follow-up 10.7 months**	**96.7**	**90**	**100**	**n**	**43.3**	[59]
Wen Y, 2025	Chidamide and CAG (cytarabine, aclarubicin, G-CSF)	34 Phase I	1-year OS 86.5%	n	n	n	n	n	[60]

## Data Availability

No new data were created or analyzed in this study. Data sharing is not applicable to this article.
